# First person – Nouf Khan and Talhah Mohd Salmi

**DOI:** 10.1242/dmm.052159

**Published:** 2024-11-25

## Abstract

First Person is a series of interviews with the first authors of a selection of papers published in Disease Models & Mechanisms, helping researchers promote themselves alongside their papers. Nouf Khan and Talhah Mohd Salmi are co-first authors on ‘ Optimized methods to image hepatic lipid droplets in zebrafish larvae’, published in DMM. Nouf is a research assistant in the lab of Kirsten C. Sadler at New York University Abu Dhabi, Abu Dhabi, United Arab Emirates, investigating the function of activating transcription factor (Atf6) in the context of development and stress response in zebrafish. Talhah conducted the research described in this article while a postdoctoral researcher in the lab of Associate Professor Andrew Cox at Peter MacCallum Cancer Centre, Melbourne, Australia, and is now an In-House Clinical Research Associate at Icon Biotech, South Yarra, Australia, investigating how oncogenic transcription factors regulate metabolism to promote liver cancer.


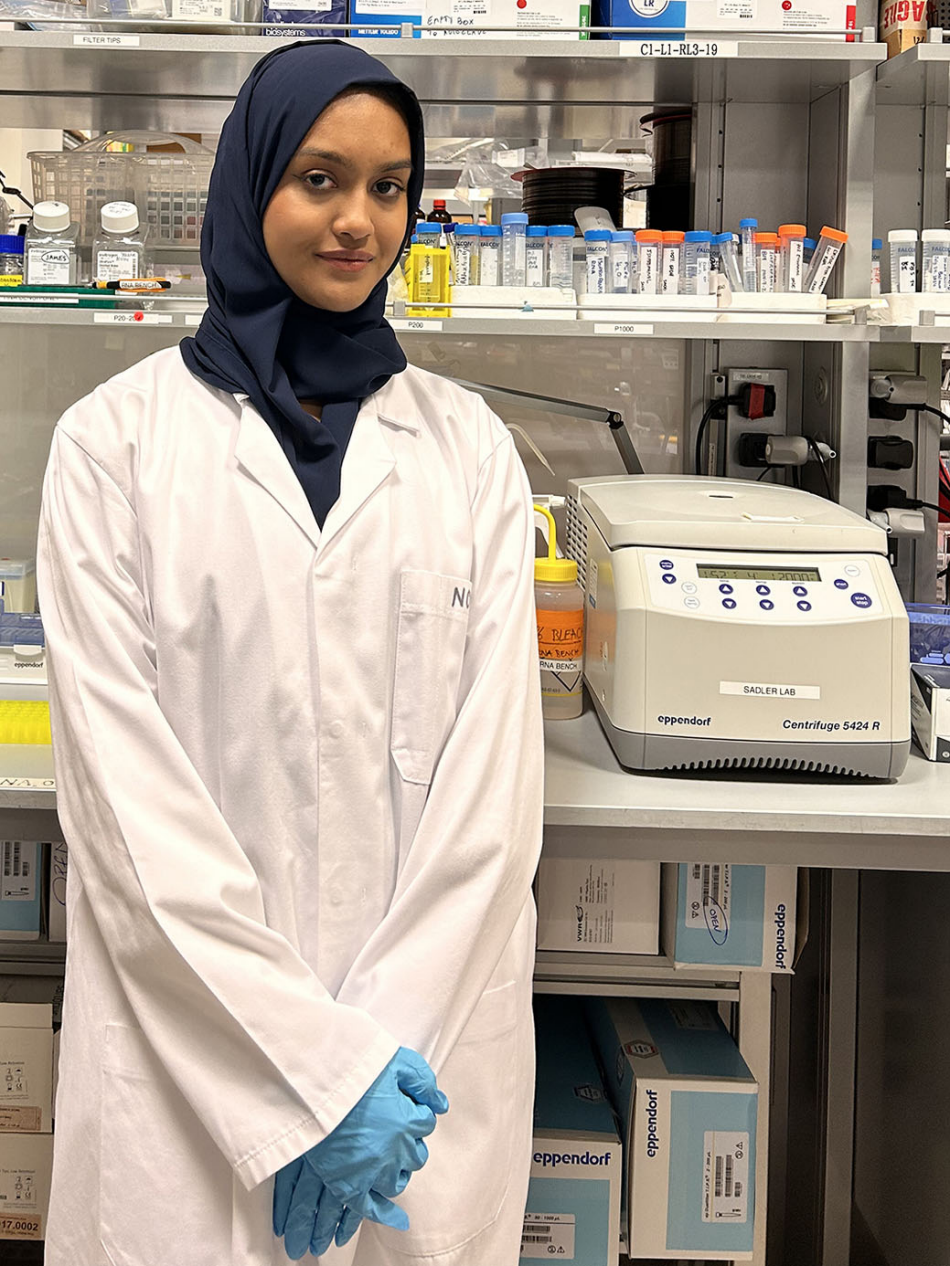


**Nouf Khan**

**Who or what inspired you to become a scientist?**

**N.K.:** My interest in biology began in high school when my teacher allowed us to dissect a liver to investigate its physiological components. While most of my classmates found it grotesque, I became increasingly curious about the intricate cellular and molecular structures of the organ. Initially, I aimed to pursue a career in medicine, but, during my sophomore year of college, my supervisor, Patrice Delaney, introduced me to my first zebrafish embryo. I was captivated by its structure and became determined to explore various aspects of developmental and molecular biology. My fascination with the liver has grown for many reasons, and my training at the Sadler Lab has demonstrated the value of curiosity in research. As I embark on my career, being surrounded by exceptional scientists, particularly my principal investigator, Kirsten Sadler, who has mentored me through both the challenges and triumphs of science, has only deepened my passion for biology and research.

**T.M.S.:** My dad has always been my idol, and his passion for teaching and research inspired me to pursue my own path in the research field. As a university lecturer and researcher in mathematics, he devoted his life to sharing knowledge, igniting curiosity in countless students. Additionally, his enthusiasm in research and discovery made science feel like a thrilling adventure. Watching him inspire others reinforced my desire to follow in his footsteps to becoming a scientist.

We showed through a comparative approach the best methods to measure lipid diameter and lipid accumulation to analyse steatosis in zebrafish larvae.

**What is the main question or challenge in disease biology you are addressing in this paper? How did you go about investigating your question or challenge?**

**N.K.:** Our study investigates a myriad of dyes and trackers available to observe and visualize lipid droplets in zebrafish larvae in the context of fatty liver disease. To quantify lipid droplets, we used traditional staining methods such as histology and Oil Red O; dyes such as Nile Red, LipidSpot and LipidTox; and a transgenic lipid marker line. We showed through a comparative approach the best methods to measure lipid diameter and lipid accumulation to analyse steatosis in zebrafish larvae. This study can serve as a great resource for other researchers hoping to find the best lipid staining method for their respective experiments.

**T.M.S.:** The main purpose of this Resources & Methods paper is to showcase different approaches by which cellular lipid droplets can be visualized in the liver of zebrafish. We utilized the zebrafish as an animal model owing to their transparent embryos, which allow for advanced microscopy techniques to image hepatic lipid droplets in live or fixed tissue. We employed multiple lipid staining techniques and took advantage of transgenic zebrafish lines, and demonstrated the strengths and weaknesses of each approach. We and others have shown how the dysregulation of lipid metabolism can contribute to liver hyperplasia. It is hoped that the methods presented in our paper will aid other researchers to gain further understanding into the role of lipid droplets in both developmental and disease biology settings.


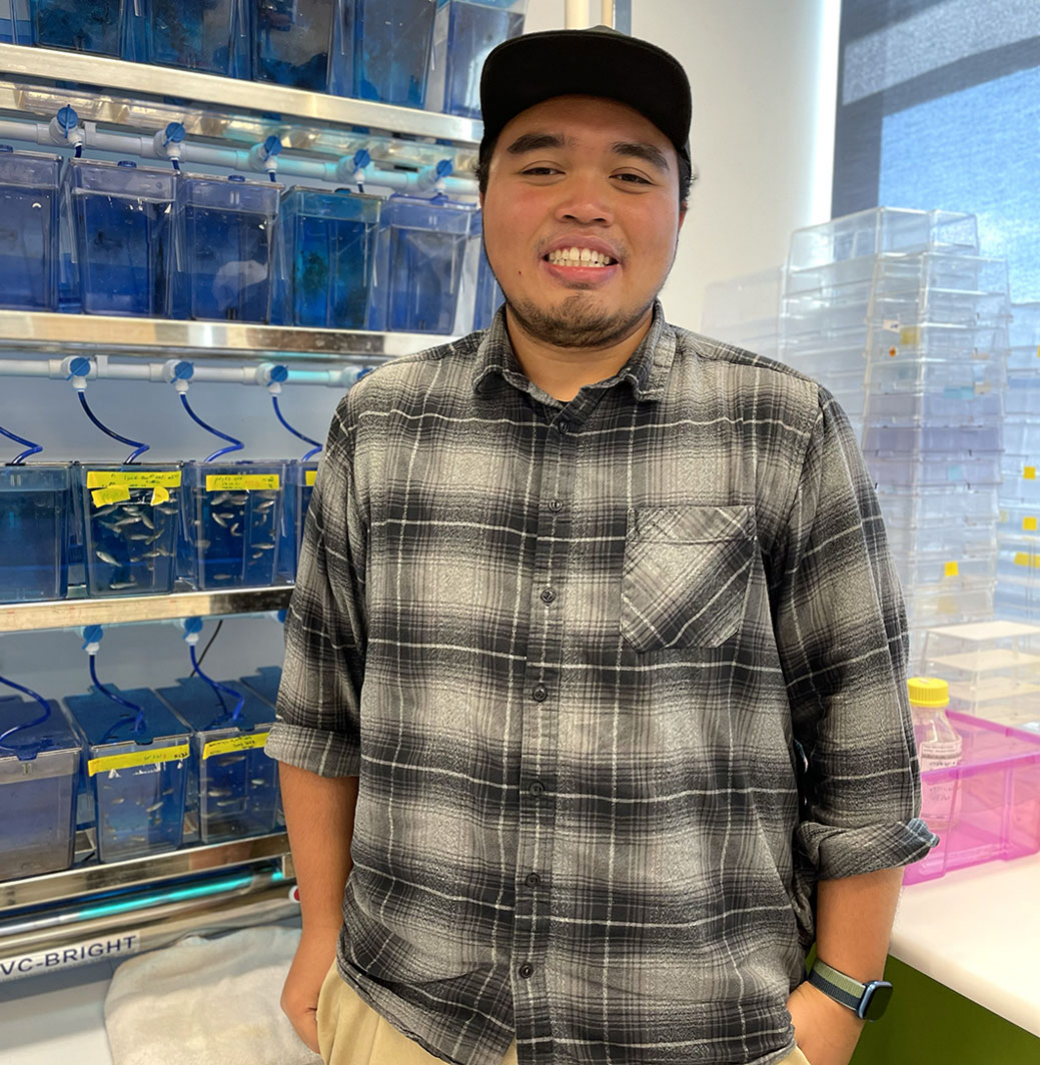


**Talhah Mohd Salmi**

**How would you explain the main findings of your paper to non-scientific family and friends?**

**N.K.:** In this study, we decided to use zebrafish to observe lipid droplets because it has a transparent embryo, which allows us to observe the different early developmental changes. This paper will serve as a reference to the various ways to mark these lipids so scientists can visualize and quantify them for the purpose of studying fatty liver disease.

**T.M.S.:** In our paper, we used zebrafish larvae as an animal model to study lipid storage in the liver in the context of development and disease. Because the zebrafish larvae are optically transparent, we can visually observe the lipids in their livers using some special fluorescent dyes and genetic engineering coupled with sophisticated microscopy. Taking advantage of this unique feature, we reviewed different ways to visualize and quantify these lipid droplets in the zebrafish liver. The aim of this paper was to provide useful tools for other researchers using the zebrafish to study the role of lipids in both organ development and disease.

**What are the potential implications of these results for disease biology and the possible impact on patients?**

**N.K.:** This paper will be a great resource to investigate lipid droplets in zebrafish larvae in the context of fatty liver disease. The zebrafish community can truly benefit from this paper because it provides a comparison between all the available dyes and trackers to stain lipid droplets by considering multiple technical variables such as sensitivity in detecting, types of microscope used, affordability of materials, and versatility of live versus fixed imaging.

**T.M.S.:** The involvement of lipid metabolism in liver pathogenesis is gaining attention in the scientific community. In my former laboratory, we showed how an oncogenic transcription factor was able to regulate *de novo* lipogenesis to promote hyperplastic liver growth. With the detailing of multiple approaches by which hepatic lipid droplets could be imaged *in vivo*, we hope that these could attract more researchers to study fatty liver disease like non-alcoholic steatohepatitis.

**Figure:**
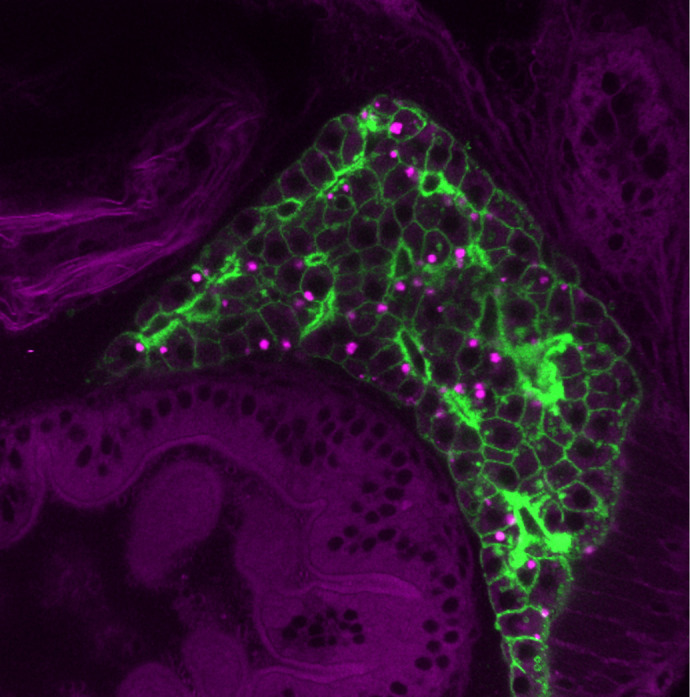
**A *tg(fabp10a;CAAX-EGFP)* zebrafish larva at 7 days post fertilization, stained with Nile Red to mark lipid droplets (magenta), with GFP-marked hepatocytes in the liver.** This sample was fixed and imaged with confocal microscopy.

**Why did you choose DMM for your paper?**

**N.K.:** We chose DMM because it is an excellent journal to highlight the significance of this methods paper. DMM provides strong support for showcasing the diverse applications of various animal models, and since this study utilized the zebrafish model to investigate lipid droplets, we couldn't think of a more suitable journal. As this resource becomes more widely available, we hope that other researchers will find it useful and valuable for designing their future experiments.

**T.M.S.:** We picked DMM because it is a prestigious developmental biology journal that provides a great platform for highlighting our methods paper. We know that DMM focuses on the use of animal models like the zebrafish, which are widely used in developmental biology and disease studies. By publishing our work here, we hope to reach researchers with the same interests and apply the methods we presented in their own work.

With the numerous advantages offered by industry or tech jobs, choosing an academic path becomes a challenging decision.

**Given your current role, what challenges do you face and what changes could improve the professional lives of other scientists in this role?**

**N.K.:** As someone currently applying to graduate school, my biggest concern is the instability associated with a career in academia. With the numerous advantages offered by industry or tech jobs, choosing an academic path becomes a challenging decision. The uncertainty of constantly applying for grants to sustain one's career, along with short-term positions that often lack professional or financial stability, can be daunting for emerging researchers.

**T.M.S.:** After leaving academia to start a career in clinical trials, I am learning how to overcome some new challenges in this industry. Transitioning from the academic research, where curiosity, exploration and long-term projects are emphasized, to the fast-paced clinical trials industry pushes me to be more adaptable and make important decisions quickly. Instead of working with other researchers, I now work with hospital staff that conduct clinical trials sponsored by different pharmaceutical companies of varying disease background.

With my new role in clinical trials, I could potentially improve the professional lives of scientists by fostering a culture of collaboration and open communication between basic science researchers and clinical science researchers.

**What's next for you?**

**N.K.:** I am currently completing my final year as a research assistant as part of the Kawader research fellowship program at the Sadler Lab, and I am preparing my application to graduate school. I am hoping to go into biomedical studies to specialize in endocrinology or reproductive science.

**T.M.S.:** I am building a career in clinical trials where I hope to gain more understanding of science, particularly in translational medicine. My aim is to serve a bridge between basic science and translational research, helping the movement of discoveries from the wet lab to clinical applications. In the future, I want to work closely with researchers and clinicians to ensure that innovative findings are effectively translated into treatments that can benefit patients in need. Ultimately, I aspire to contribute to developing therapies that address unmet clinical needs, making significant impact on patients' lives through my work in clinical trials.

**Tell us something interesting about yourself that wouldn't be on your CV**

**N.K.:** I have a deep appreciation for the beauty of the miniscule, and, had I not become a scientist, I likely would have pursued a career as an artist, spending my days fabric painting and embroidering amorphous patterns on various mediums. I feel fortunate to have conducted many imaging-based experiments, as I am a very visual person. Capturing lipid droplets in hepatocytes with different dyes often felt like an artistic endeavour, which made the experience even more enjoyable.

**T.M.S.:** I really love travelling, and it's a passion that has enriched my life in so many ways. Exploring new cultures and landscapes not only offer me a break from my usual routine, but also inspires my life and work. Each place I visit teaches me something different, whether it's trying local cuisine, learning a new language or understanding unique perspectives. This not only broadens my horizons but also helps me build a global network, allowing me to bring back fresh ideas and insights. Travelling has truly shaped my outlook on life, making me adaptable and open-minded.
